# Cyclodextrins as eminent constituents in nanoarchitectonics for drug delivery systems

**DOI:** 10.3762/bjnano.14.21

**Published:** 2023-02-09

**Authors:** Makoto Komiyama

**Affiliations:** 1 Research Center for Advanced Science and Technology (RCAST), The University of Tokyo, 4-6-1 Komaba, Meguro, Tokyo 153-8904, Japan

**Keywords:** cyclodextrin, drug delivery system (DDS), nanoarchitecture, phototherapy, siRNA

## Abstract

Cyclodextrins have been widely employed for drug delivery systems (DDSs) in which drugs are selectively delivered to a target site in the body. Recent interest has been focused on the construction of cyclodextrin-based nanoarchitectures that show sophisticated DDS functions. These nanoarchitectures are precisely fabricated based on three important features of cyclodextrins, namely (1) the preorganized three-dimensional molecular structure of nanometer size, (2) the easy chemical modification to introduce functional groups, and (3) the formation of dynamic inclusion complexes with various guests in water. With the use of photoirradiation, drugs are released from cyclodextrin-based nanoarchitectures at designated timing. Alternatively, therapeutic nucleic acids are stably protected in the nanoarchitectures and delivered to the target site. The efficient delivery of the CRISPR-Cas9 system for gene editing was also successful. Even more complicated nanoarchitectures can be designed for sophisticated DDSs. Cyclodextrin-based nanoarchitectures are highly promising for future applications in medicine, pharmaceutics, and other relevant fields.

## Review

### 1 Introduction

Recently, drug delivery systems (DDSs) have been attracting much interest [1–12]. By delivering drugs selectively to disease sites, the drug dosage can be reduced, and undesired side-effects are minimized. Undoubtedly, these methodologies are essential for further progresses in medicine. In order to accomplish eminent DDS, drugs should be combined with appropriate carriers to fulfill several requirements. First, the composite needs be sufficiently stable in the body and successfully reach the target site without being trapped. Second, the composites must be efficiently internalized to the target tissues and cells. Finally, the drugs must be released there at desired timing or be activated to medicinally active species. As the carriers for DDSs, nanoarchitectures are very powerful and convenient since all the required properties can be easily provided through detailed molecular design [1,13]. Furthermore, the encapsulated drugs can be promptly released, when necessary, by disassembling the nanoarchitectures completely (or partially) at desired timing. Recent progresses in this research field have been remarkable, resulting in notable improvements in clinical performance.

In order to construct sophisticated nanoarchitectures, cyclodextrins (CyDs) are especially promising as essential components [14–34]. The most attractive feature of these cyclic oligosaccharides is their discrete molecular structure, which functions as unique molecular-sized framework to construct a variety of nanostructures. The cylindrical structures are stabilized by rings of intramolecular hydrogen bonds between adjacent glucose units. The internal diameters of the cavities of α-, β-, and γ-CyDs (composed of six, seven, and eight ᴅ-glucose units) are about 4.5–6, 6–8, and 8–9.5 Å, respectively. The inside of the cavity is apolar in nature, and thus apolar guests of suitable size are preferentially accommodated to form inclusion complexes. The CyD/guest ratio in inclusion complexes is usually 1:1. Very importantly for practical applications in DDSs, CyDs show no toxicity to the human body since they are enzymatic digests of starch. By various chemical modifications, the desired number and kind of functional groups can be easily and precisely introduced to predetermined sites, greatly widening the scope of applications [35–37]. In pharmaceutics, CyDs (and their derivatives) solubilize poorly water-soluble drugs through encapsulation in the cavity. Furthermore, otherwise unstable drugs are protected from degradation for efficient therapeutic functions. Many other roles of CyDs are also evident. Advantageously for DDSs, inclusion complexes of CyDs are reasonably stable to deliver drugs to the target site but can be rather easily dissociated at the target site through a small perturbation. Hence, by applying photoirradiation, temperature increase, pH change, or other stimuli to CyD-based nanoarchitectures, the encapsulated drug can be released at required timing for on-demand therapeutics.

**Figure 1:**
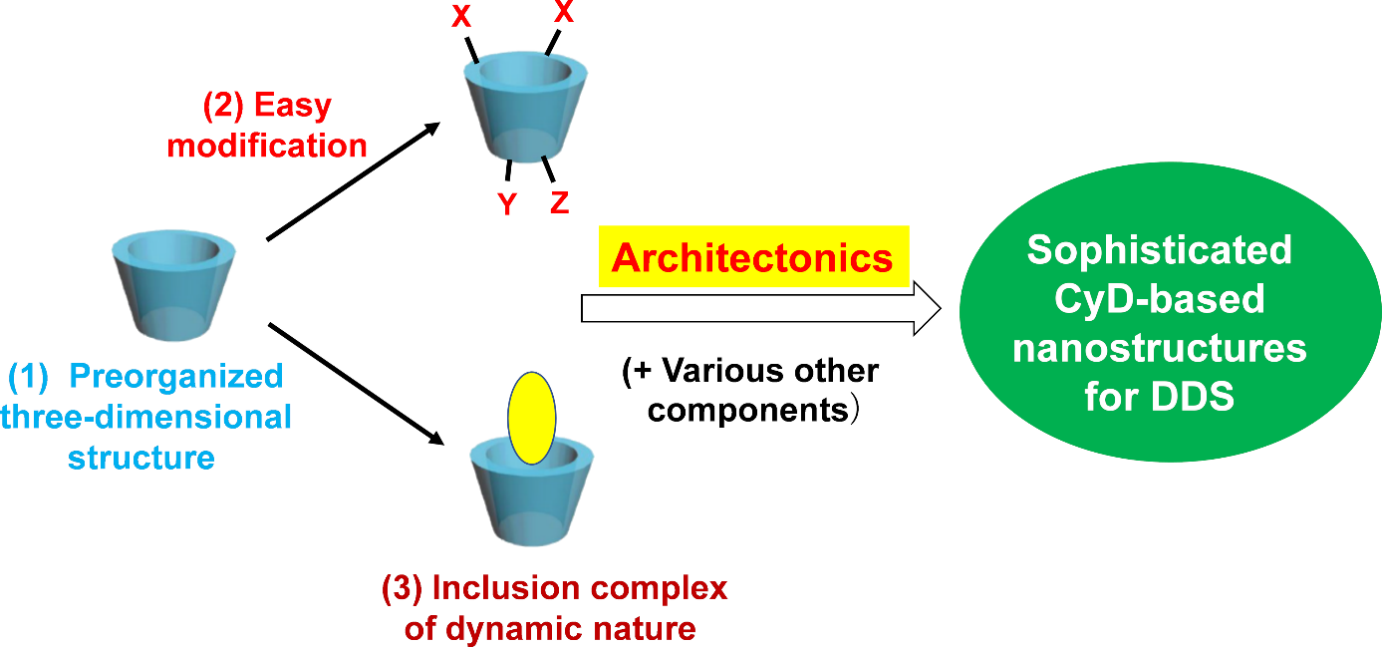
Three essential features of CyDs to construct sophisticated nanoarchitectures for DDS.

In the early stage of pharmaceutical applications, CyDs were mostly employed in their monomeric forms or as their simple aggregates [14]. The inclusion complexes with drugs were usually prepared by mixing the components in water. When necessary, CyDs were crosslinked and used in the forms of, for example, hydrogels and nanoparticles [38–46]. More recently, however, precisely designed CyD-based nanoarchitectures are primarily considered since more sophisticated and complicated functions are designable and accomplishable (Figure 1). The construction of sophisticated nanoarchitectures is greatly facilitated by three important features of CyDs, namely (1) the preorganized three-dimensional molecular structure of nanometer-size, (2) the easy chemical modification to introduce functional groups, and (3) the formation of dynamic inclusion complexes with various guests in water. The CyDs in nanoarchitectures mostly behave like monomeric molecules, and new functions are additionally envisioned in nanoarchitectonics.

This review will cover recent developments of CyD-based nanoarchitectures for advanced DDSs. Essential roles of CyDs for the construction of eminent nanoarchitectures are presented with the emphasis on the three features of CyDs in Figure 1. First, the photoinduced delivery of chemical drugs by CyD-based carriers is described (section 2). The dynamic features of CyD inclusion complexes are essential here since minor changes of the molecular structure of chromophores, induced by photoirradiation, ultimately decompose the whole nanoarchitecture for drug release. In section 3, various therapeutic nucleic acids are delivered to the target site by CyD nanoarchitectures. By embracing with chemically modified CyDs, otherwise fragile nucleic acids are successfully delivered to the target site and suppress the designated gene expression. Furthermore, various CyD-based photoinduced therapies (e.g., photodynamic and photothermal therapies) are described in sections 4 and 5. Finally, some of the recent topics of CyD-based DDSs will be presented in section 6. A comprehensive survey of applications of CyDs in DDSs is beyond this review, and the readers who are interested in them should refer to other excellent reviews and articles [14–34].

### 2 CyD-based nanoarchitectures for photoresponsive delivery of chemical drugs

Light irradiation is an eminent stimulus for the on–off control of drug delivery since light can be strictly focused to target sites and irradiated only when necessary. These two factors facilitate the precise spatiotemporal control of the therapy and minimize undesired side-effects. Most photoresponsive DDSs based on CyD-based nanoarchitectures take advantage of structural changes of appropriate chromophores. Upon photoirradiation inclusion complexes of CyDs are dissociated, inducing the breakdown of CyD-based nanoarchitectures to release the encapsulated drugs. The isomerization of azobenzene or its analogues is the most widely employed. CyD-based nanoarchitectures are sufficiently dynamic so that even these subtle molecular changes induce the dissociation of the whole nanoarchitecture for the release of the encapsulated drug.

#### 2.1 Drug release from CyD-based nanoarchitectures through the isomerization of azobenzene

Azobenzene isomerizes upon UV irradiation (around 300 nm) from the *trans* form to the *cis* form [47]. The reverse isomerization (from *cis* to *trans*) is accomplishable by light with longer wavelength (e.g., 400 nm). This isomerization is a notable structural change of the molecule and, thus, very useful to regulate the formation/deformation of supramolecular nanoarchitectures. Importantly, only *trans*-azobenzene can form inclusion complexes with CyD, since it has a straight and flat structure that fairly fits the cavity of CyD. In contrast, the *cis* isomer is too bulky (and non-planar) and hardly accommodated by the cavity. In many cases, the photoisomerization of azobenzene can proceed smoothly even in nanoarchitectures. For example, photoresponsive supramolecular nanoparticles were prepared from azobenzene-modified β-CyD and a polymer involving α-CyD units [48]. Prior to photoirradiation, azobenzene takes the *trans* form, and is included into the cavity of α-CyD to form nanoparticles. Upon irradiation with UV light, however, azobenzene isomerizes to the *cis* form, leading to the breakdown of the inclusion complex with α-CyD. Thus, the nanoparticles are disassembled to release the encapsulated drugs.

β-CyD units have been connected to mesoporous silica nanoparticles via a disulfide linkage, and azobenzene molecules have been bound to a galactose-grafted polymer [49]. Prior to photoirradiation, the *trans* isomer of azobenzene forms an inclusion complex with β-CyD bound to the silica nanoparticles and seals the pores of nanoparticles in which drugs are encapsulated. Under these conditions, the composite is stable, because azobenzene is accommodated in the cavity of β-CyD, and sterically protects the disulfide linkage from reductive cleavage by endogenous glutathione, which is abundant in the cells. Upon UV irradiation, however, azobenzene isomerizes from the *trans* form to the *cis* form and spontaneously dissociates from β-CyD. As a result, the disulfide linkage becomes more exposed and is reductively cleaved by glutathione. Thus, the mesopore is opened for the release of encapsulated drug.

**Figure 2:**
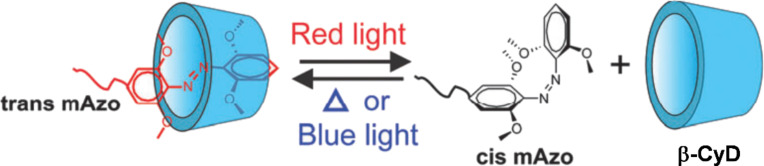
Visible-light responsive isomerization of tetra-*ortho*-methoxy-substituted azobenzene (mAzo) to regulate the formation of inclusion complexes with β-CyD. Figure 2 was reproduced from [50] (“Supramolecular hydrogels constructed by red-light-responsive host–guest interactions for photo-controlled protein release in deep tissue“ by D. Wang et al., © The Royal Society of Chemistry 2015, distributed under the terms of the Creative Commons Attribution 3.0 Unported License).

In order to achieve photoinduced drug release through visible light, tetra-*ortho*-methoxy-substituted azobenzene (mAzo) can be used instead of unsubstituted azobenzene. The *trans*-to-*cis* isomerization of this chromophore proceeds under irradiation with red or green light, and the reverse *cis*-to-*trans* isomerization occurs under blue light [50]. Compared with UV light, visible light is advantageous for medical applications, since it can penetrate more deeply into tissues and causes less photodamage to healthy cells. In the same way as unsubstituted azobenzene, only the *trans* isomer of mAzo efficiently forms inclusion complexes with β-CyD (Figure 2). The surface of mesoporous silica nanoparticles was modified with β-CyD and covered with a polymer bearing mAzo units [51]. Drugs were loaded in the pores of the silica nanoparticles. Prior to photoirradiation, the *trans* isomer of mAzo on the polymer was efficiently included into the cavity of β-CyD and functioned as gatekeeper of the pores for the stable encapsulation of the drugs. Upon irradiation with green light, however, mAzo switched from the *trans* form to the *cis* form to break down the inclusion complex. Accordingly, the gate was opened, and the loaded drugs were released.

#### 2.2 More efficient photoinduced drug release using arylazopyrazole

**Figure 3:**
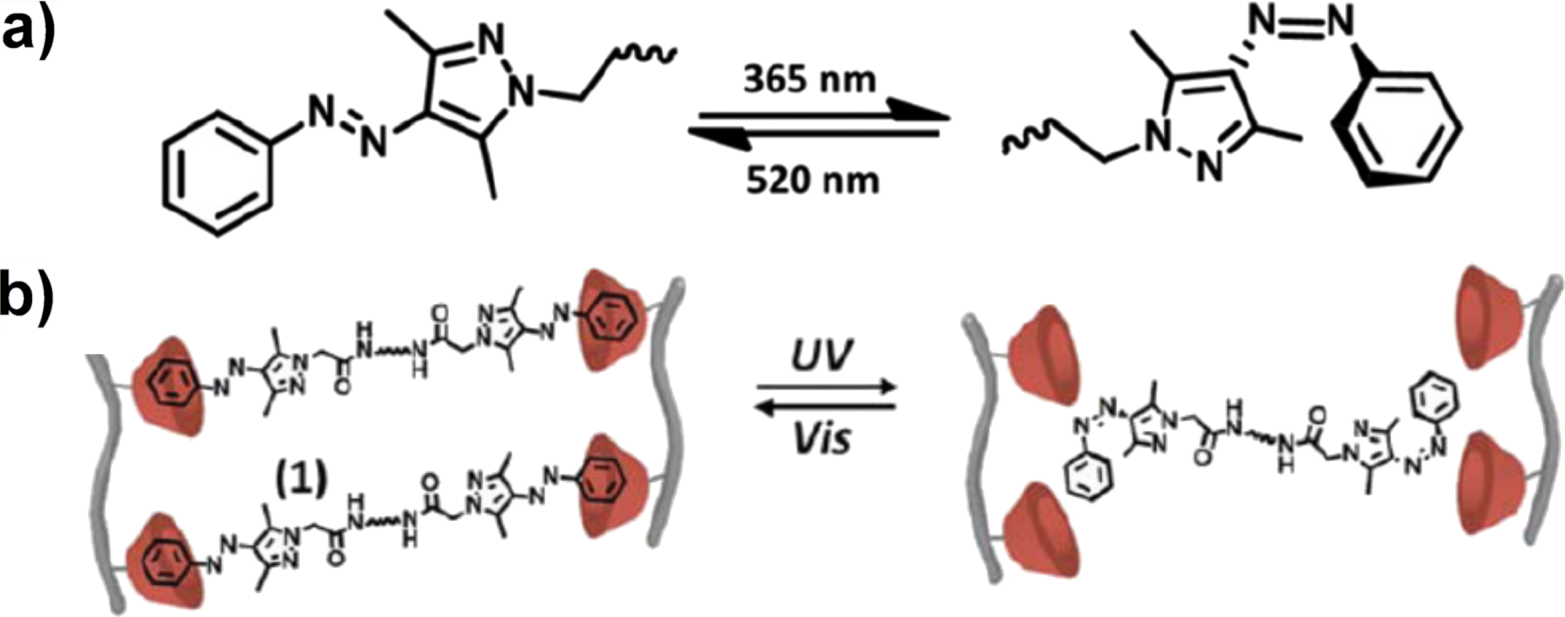
(a) Photoisomerization of arylazopyrazole. Note that the photoisomerization is essentially complete (100% conversion) in both directions. (b) Reversible gel–sol transition by using on–off photoisomerization of arylazopyrazole between the *trans* form and the *cis* form. Figure 3b was used with permission of The Royal Society of Chemistry from [53] (“Light-responsive arylazopyrazole-based hydrogels: their applications as shape-memory materials, self-healing matrices and controlled drug release systems” by G. Davidson-Rozenfeld et al., *Polym. Chem.*, vol. 10, issue 30, © 2019); permission conveyed through Copyright Clearance Center, Inc. This content is not subject to CC BY 4.0.

As described above, the light-induced *trans*/*cis* photoisomerization of azobenzenes is a very useful tool to construct photo-switchable nanoarchitectures. However, its main drawback is that the isomerizations never reach 100% conversion (the *trans*/*cis* ratio in the photo-equilibrium is around 2:8). Thus, a complete on–off photoregulation is hardly accomplishable with azobenzenes. To solve this problem, arylazopyrazoles (Figure 3a) are very convenient. They have smaller band overlap of the *trans*/*cis* isomers, and thus the photoisomerization proceeds almost completely in both directions. Moreover, thermal relaxation of the cis isomer is negligible [52]. Like azobenzenes, only the *trans* isomer forms inclusion complexes with CyDs. In Figure 3b, carboxymethyl cellulose chains were functionalized with β-CyD, and hydrogels were prepared by adding connector molecules bearing two arylazopyrazole groups at the ends [53]. Without photoirradiation, the arylazopyrazoles are stably accommodated in the cavities of β-CyD groups, and thus the gel is well crosslinked and very stiff. Upon photoirradiation, however, the crosslinkings for the gel formation are broken almost completely, releasing the encapsulated drugs (e.g., DOX as anticancer drug).

#### 2.3 Upconversion nanoparticles to release drugs by near-infrared light

As an external stimulus for DDSs, near-infrared (NIR) light is more advantageous than UV and visible light, since it penetrates more efficiently into tissues, and the toxicity is marginal. However, most photoisomerizable dyes (azobenzenes and arylazopyrazoles) require UV or visible light and are incompatible with NIR. The most straightforward strategy is the use of upconversion nanoparticles to convert NIR to shorter-wavelength light for photoreactions. These nanoparticles usually contain rare-earth metal ions, which have long-living excited states. Thus, they achieve sequential energy absorption and convert two or more low-energy (NIR) photons into UV or visible light [54]. In a pioneering work [55], α- and β-CyDs were bound to the surface of upconversion nanoparticles and either azobenzene or arylazopyrazole was added to form inclusion complexes. NIR irradiation (980 nm) was converted to shorter-wavelength light, which isomerized azobenzene or arylazopyrazole to the *cis* form. Accordingly, the inclusion complexes were spontaneously broken down.

NaYF_4_:Yb,Tm@NaLuF_4_ upconversion nanoparticles were used for DDSs [56]. The surface of these nanoparticles was covered with a mesoporous silica layer, and drugs were encapsulated in the mesopores. To the mesoporous silica layer, pyrene molecules were connected by ester linkage, and β-CyD was added to form inclusion complexes with them. The inclusion complex was sufficiently bulky to block the release of drugs from the pores. NIR irradiation (980 nm) was converted to 340 nm light (and 475 nm light) by the upconversion nanoparticles, which in turn cleaved the covalent linkages between pyrene molecules and the mesoporous silica layer. As the result, the pores were unblocked and the encapsulated drugs were released.

### 3 CyD-based nanoarchitectures to deliver therapeutic nucleic acids to the target site

Therapeutic nucleic acids are RNAs, DNAs, and derivatives thereof that regulate the expression of target genes [57–59]. Typical examples are antisense DNA, small interfering RNA (siRNA), aptamers, and ribozymes/DNAzymes. Owing to the accurate targeting at pathogenic genes, they are promising for various applications. However, nucleic acids are intrinsically unstable in serum and do not readily cross the cellular plasma membranes. Accordingly, it is a big challenge to deliver them in the intact form into target cells. Conventional gene transfection reagents are not very good candidates, since multiple positive charges of high density can damage the membranes and organelles of normal cells. With the use of CyD-based DDSs, however, high transfection efficiency and low cytotoxicity have been accomplished with minimal immune stimulation. The preorganized three-dimensional molecular structure of CyD as well as the resultant well-organized orientation of functional groups in CyD-based nanoarchitectures, should be very appropriate to yield both stable encapsulation of nucleic acid drugs and their prompt release when needed.

#### 3.1 CyD-based nanoarchitectures for delivery of siRNA

The therapeutic nucleic acid siRNA is double-stranded RNA of 21–23 base-pair length and a powerful tool to suppress the expression of target genes [60–61]. However, siRNAs are easily digested by nucleases in our body and difficult to deliver to target cells without appropriate tactics. The use of CyD-based nanoarchitectures as vehicles is an eminent solution to this problem. For example, polyamidoamine dendrimer (generation 3) conjugated with α-CyD (average number = 2.4) is very effective to deliver siRNA to cells [62–63]. By appending folate, the conjugates are successfully targeted to tumor cells on which folate receptors are abundantly expressed. In the positively charged dendrimer the siRNA is stably protected from enzymatic digestion. The composite easily escapes from the endosome through the proton sponge effect of the dendrimer. Furthermore, inclusion complex formation of α-CyD with phospholipids facilitates the release of siRNA from the endosome. In another example, a supramolecular nanoparticle was prepared from a linear CyD-based polymer, hydrophilic polyethylene glycol bearing an adamantane at the end, and siRNA [64]. By attaching a human transferrin protein, this composite was steered to target cancer cells to suppress the expression of the target gene. A polymer bearing cationic CyD was also effective in delivering siRNA [65].

#### 3.2 Photoinduced release of siRNA from CyD-based nanoarchitectures

**Figure 4:**
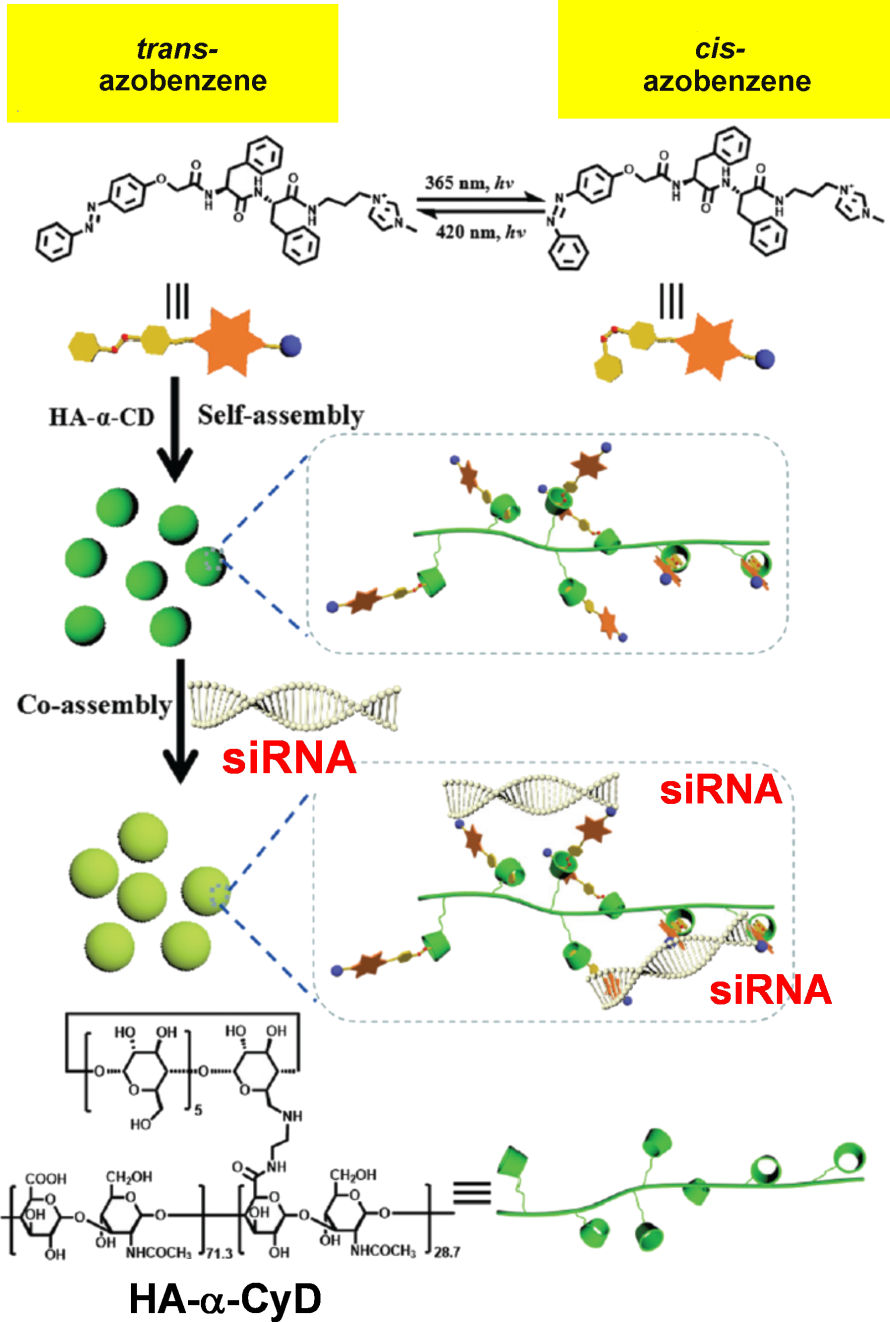
Photoinduced delivery of siRNA by a composite formed from α-CyD-modified hyaluronic acid (HA-α-CyD) and an azobenzene-modified diphenylalanine derivative. Upon UV irradiation, azobenzene isomerizes from the *trans* form to the *cis* form, decomposing the composite to release the siRNA cargo. Figure 4 was adapted with permission of The Royal Society of Chemistry from [66] (“Highly efficient photocontrolled targeted delivery of siRNA by a cyclodextrin-based supramolecular nanoassembly” by F.-Q. Li et al., *Chem. Commun.*, vol. 56, issue 27, © 2020); permission conveyed through Copyright Clearance Center, Inc. This content is not subject to CC BY 4.0.

The photoisomerization of azobenzene (section 2.1) is useful to release siRNA cargo from CyD-based nanoarchitectures at the target site with desired timing. In Figure 4, hyaluronic acid (HA) is modified with α-CyD (green torus), and diphenylalanine is modified with both azobenzene (golden hexagons/small red balls) and *N*-methylimidazolium ions (small dark blue circle) [66]. From these two components, supramolecular nanoassemblies (green balls) were constructed through inclusion complex formation between *trans*-azobenzene and α-CyD (the second row from the top). These α-CyD-based nanoassemblies bind siRNA through electrostatic interactions with the positively charged *N*-methylimidazolium ions to form ternary supramolecular nanoassemblies (pale green balls in the third row from the top). Due to high biocompatibility and tumor-targeting capacity of HA, these ternary nanoassemblies effectively entered cancer cells. Upon UV irradiation (365 nm), the azobenzene isomerizes from the *trans* form to the *cis* form, disassembling the α-CyD inclusion complex (thus, the ternary assembly is also disassembled). As the result, the siRNA cargo is released and shows excellent cytotoxicity against cancer cells.

**Figure 5:**
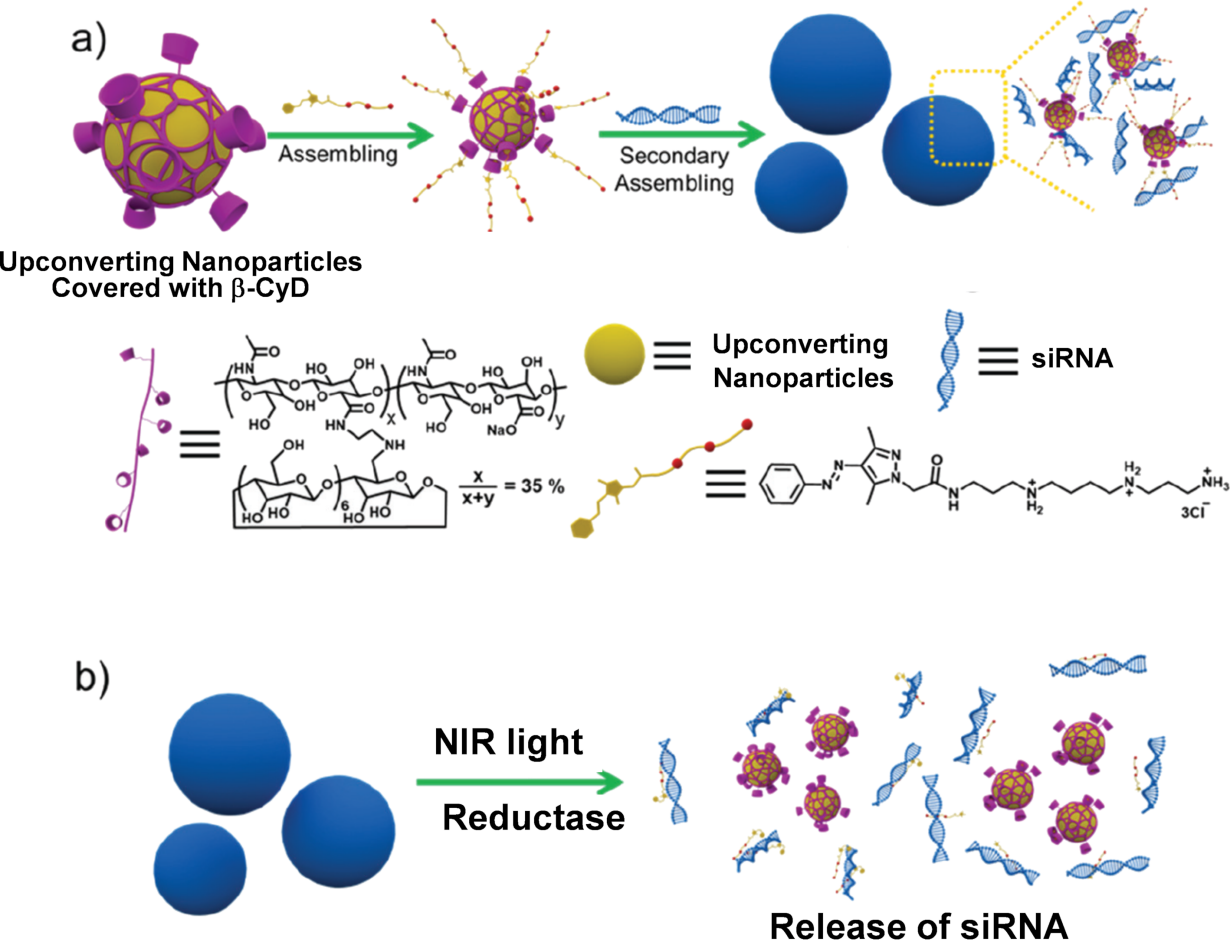
Release of siRNA responding to the irradiation of NIR as an external stimulus. By the upconversion particles (yellow balls), the NIR light is converted to the light of shorter wavelength, which is absorbed by the arylazopyrazole. Resultant isomerization of this dye from the *trans* form to the *cis* form breaks down the β-CyD inclusion complex, and induces the decomposition of nanocomposites (blue balls) to release the siRNA. Figure 5 was adapted with permission of The Royal Society of Chemistry from [67] (“Highly effective gene delivery based on cyclodextrin multivalent assembly in target cancer cells” by Y.-H. Liu and Y. Liu, *J. Mater. Chem. B*, vol. 10, issue 6, © 2022); permission conveyed through Copyright Clearance Center, Inc. This content is not subject to CC BY 4.0.

In Figure 5, NIR light is used (instead of UV in Figure 4) to release siRNA at the target site. As described in section 2.3, the NIR irradiation is converted by upconversion nanoparticles (yellow balls) to shorter-wavelength light, which is absorbed by arylazopyrazoles to dissociate their CyD complex (and ultimately the nanomedicine) for the release of siRNA [67]. Arylazopyrazoles are characterized by satisfactory on–off isomerization (see Figure 2). First, upconversion nanoparticles (NaYF_4_:Yb^3+^,Tm^3+^@NaYF_4_) were covered with β-CyD-containing polymer (purple). Then, spermine-modified arylazopyrazole (the *trans* form) formed a stable inclusion complex with β-CyD. When siRNA (blue helix) was added further, it was electrostatically bound to the positively charged spermine moiety to form stable nanomedicine particles (blue balls in Figure 5a). Upon irradiation of the nanocomposite with NIR light, this light was converted to UV light (around 360 nm) by the upconversion particles and absorbed by arylazopyrazole. The resultant *cis* isomer could not form inclusion complexes with β-CyD. Hence, the whole assembly spontaneously dissociated (Figure 5b). Furthermore, the azo groups were exposed to cytoplasm during the dissociation of nanoparticles and digested by the enzyme azoreductase. This unique enzyme is abundant in the cells of eukaryotes and degrades azo compounds. Because of the decomposition of nanomedicines through these two pathways, the siRNA was efficiently released in target cells and silenced the pathogenic gene.

#### 3.3 Simultaneous delivery of multiple types of therapeutic drugs

When two types of therapeutic DNA (or RNA) drugs are simultaneously delivered to a target site, the therapy should be more efficient than a unimodal therapy. For such a co-delivery, CyD-based nanoarchitectures are very convenient since well-defined structures of CyDs allow for the precise molecular design of nanoarchitectures in which the desired nucleic acid drugs are accommodated. Both antisense DNA and siRNA have been delivered by β-CyD-based nanoparticles [68]. First, two groups of seven antisense DNA strands were covalently connected to each of two β-CyD molecules. In order to bind siRNA to these antisense DNAs, each of the two strands of siRNA was extended in the 3′-direction to provide an overhang in both ends. Thus, the siRNA was captured by two kinds of antisense DNAs on the β-CyDs through RNA/DNA hybridization and co-assembled to the nanoparticles. Folic acid (for targeting tumors) and an influenza hemagglutinin peptide HA (for endosomal escape) were conjugated with adamantane and bound to these nanoparticles through non-covalent host–guest interactions between β-CyD and adamantane. When the composite entered human cells, the RNA/DNA hybrid was digested by ribonuclease H (an intracellular ribonuclease that hydrolyzes the RNA in the RNA/DNA heteroduplex) to release the siRNA and the antisense DNA strands. The two nucleic acid drugs cooperatively suppressed the expression of the target tumor gene.

**Figure 6:**
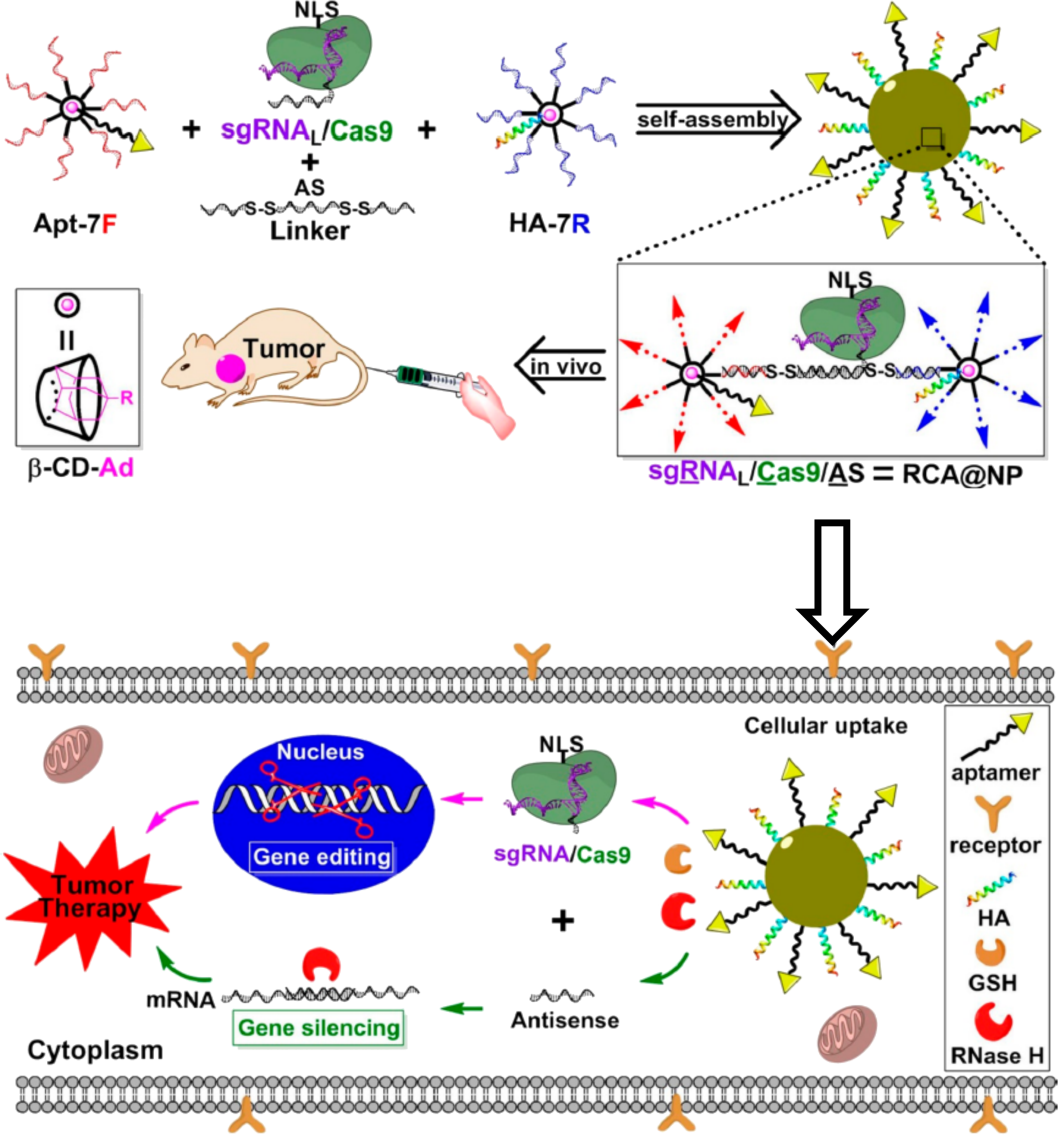
Delivery of the sgRNA/Cas9/antisense complex to tumors for synergistic therapy. 7AS1 or 7AS2, antisense DNAs covalently linked to β-CyD; sgRNA_L_, 3′-terminal-extended sgRNA. Adapted with permission from [71]. Copyright 2019 American Chemical Society. This content is not subject to CC BY 4.0.

With a similar strategy, a gene editing machine composed of Cas9 and single guide RNA (sgRNA) [69–70] was delivered to tumors simultaneously with antisense DNA (Figure 6) [71]. An sgRNA/Cas9/antisense complex was constructed to target the tumor-associated gene PLK1. Seven antisense DNA strands (AS1 in red and AS2 in blue) were bound to β-CyD, and these two products (Apt-7F and HA-7R) were connected by a linker DNA. Independently, sgRNA was extended to the 3′-end so that it binds to the linker DNA through RNA/DNA duplex formation. Two kinds of β-CyD bearing DNAs (Apt-7F and HA-7R) and the sgRNA/Cas9 composite were co-assembled to nanoparticles of 120 nm diameter (dark yellow). The composite was further modified with an aptamer (for targeting to tumors) and an influenza hemagglutinin peptide HA (for endosomal escape) by inclusion complex formation of β-CyD with adamantane. In human cells, the RNA/DNA hybrid was digested by intracellular ribonuclease H to release the antisense DNA. Furthermore, the linker is divided into three portions through the cleavage of two S–S linkages by intracellular glutathione (GSH). As the result, both gene editing by sgRNA/Cas9 and gene silencing by the antisense DNA cooperatively suppressed the PLK1 gene, providing remarkable antitumor activity.

### 4 CyD-based nanoarchitectures for effective photodynamic therapy

**Figure 7:**
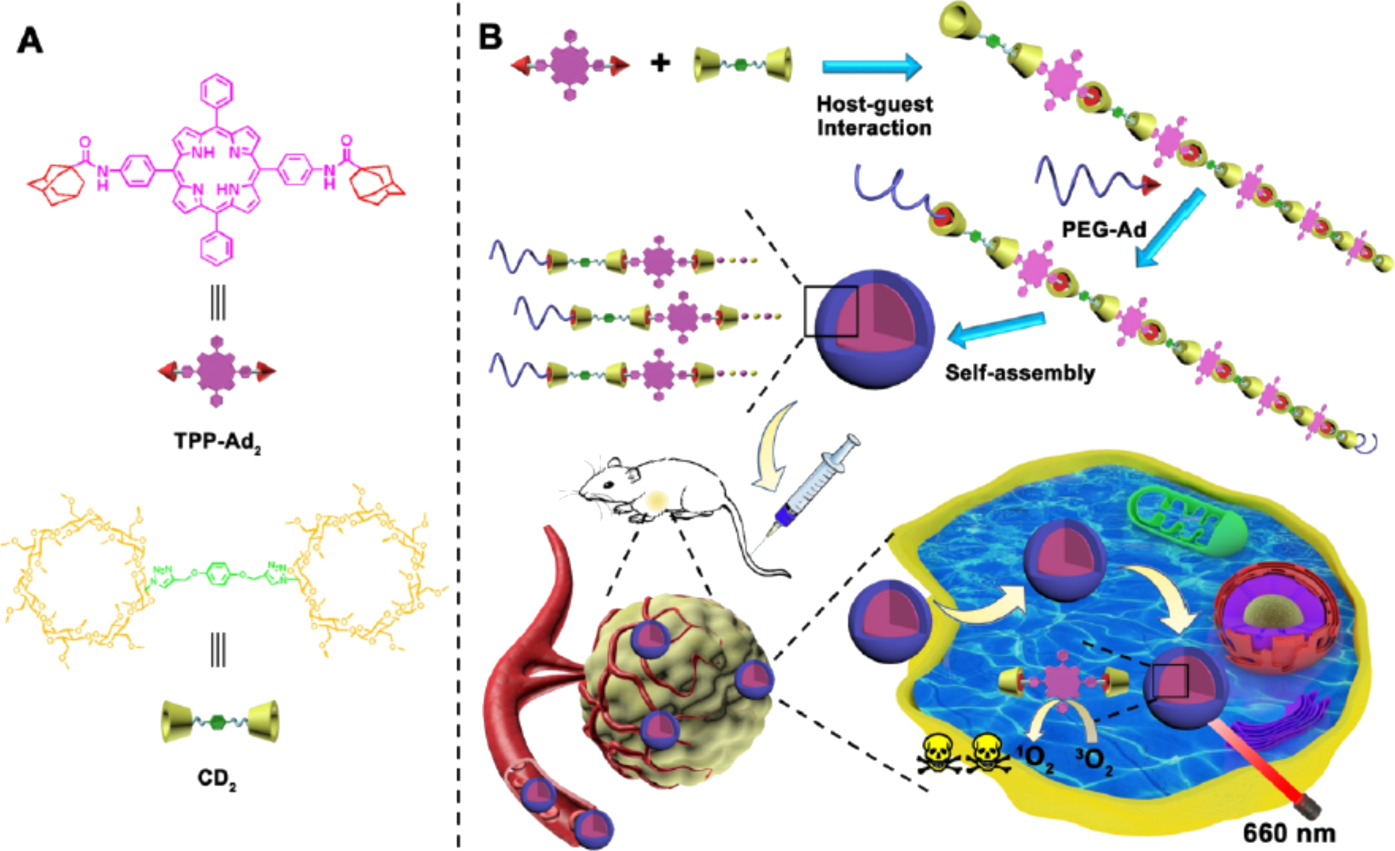
PDT by nanoparticles formed from β-CyD dimers (CD_2_), porphyrin conjugated with two adamantine molecules (TPP-Ad_2_), and poly(ethylene glycol) bearing an adamantane moiety. Adapted with permission from [74]. Copyright 2020 American Chemical Society. This content is not subject to CC BY 4.0.

Photodynamic therapy (PDT) employs a light-sensitive medicine (photosensitizer) and a light source to destroy abnormal cells [72–73]. The photosensitizer absorbs the light and is activated to kill target tissue. In many cases, reactive oxygen species (ROSs) such as singlet oxygen (^1^O_2_) generated from ^3^O_2_ by a photocatalytic process destroy subcellular structures (e.g., cell membranes or organelle membranes). Various types of cancers are effectively treatable without significant side effects. However, most photosensitizers available at present are hydrophobic and easily aggregate in aqueous solution. Thus, the efficiency of ROS production is reduced through self-quenching. In order to solve this problem, CyDs are very useful. In Figure 7A, two permethylated β-CyD molecules were connected by a linker to form a β-CyD dimer (CD_2_), whereas a porphyrin was conjugated with two adamantine molecules (TPP-Ad_2_) [74]. From them, a linear polymer was constructed through consecutive inclusion of adamantane in β-CyD units. Upon further addition of poly(ethylene glycol) bearing adamantane at the terminus nanoparticles were formed (Figure 7B). In these nanoparticles, aggregation of porphyrins is suppressed because of steric hindrance between the β-CyD units. Accordingly, ^1^O_2_ was efficiently generated by irradiation with 660 nm light since self-quenching of the photosensitizers was minimized. By using a photochromic switch moiety (two CyDs bridged with diarylethene), the efficiency of PDT was photo-regulated [75]. In order to accomplish PDT using NIR light irradiation, adamantane-modified phthalocyanine was connected to upconversion nanoparticles using carboxymethyl-β-CyD [76].

Furthermore, boron-dipyrromethene (BODIPY) photosensitizer was complexed with amphiphilic α-CyD bearing oligo(ethylene glycol) chains [77]. By encapsulating (a part of) the photosensitizer in the cavities, its self-aggregation was suppressed to accomplish a high quantum yield of ^1^O_2_ production. The aggregation of porphyrin was photocontrolled by using complex formation of β-CyD with photoresponsive azobenzene [78].

By delivering nitrogen oxide (NO) with a photosensitizer simultaneously, the efficiency of PDT is greatly improved [79]. To α-CyD, an *S*-nitrosothiol (–S–N=O) was conjugated, and another α-CyD was modified with chlorin e6 (a photosensitizer). These two modified α-CyDs were coassembled by adding a poly(ethylene glycol) derivative. Upon the entry of the resultant nanoparticles to cells, the *S*-nitrosothiol reacts with glutathione to release NO, which relieves hypoxia at tumor sites through NO-mediated blood vessel relaxation. Furthermore, the NO reacts with ROSs, generated by light-activation of chlorin e6, to form ONOO^−^ and other reactive nitrogen species that are more biocidal than ROSs. Another NO-releasing β-CyD derivative (modified with *N*-diazoniumdiolate) showed notable bactericidal activity on a Gram-negative pathogen [80]. Other NO-releasing CyDs were also reported [81–82].

In chemodynamic therapy, ROSs are produced from endogenous H_2_O_2_ by Fenton-type reactions without photoirradiation [83–84]. A nanomedicine was prepared from (i) β-CyD bearing both a ferrocene and a platinum(IV) complex (–O–Pt^IV^(Cl_2_)_2_(NH_3_)_2_(OH)) as prodrug of cisplatin and (ii) poly(ethylene glycol) terminated by ferrocene [85]. The ferrocene molecules form inclusion complexes with β-CyD to produce supramolecular nanoparticles. Upon the entry of these nanoparticles to tumor cells, ferrocene is oxidized by endogenous H_2_O_2_ into more hydrophilic ferrocenium, which is only loosely bound to β-CyD. Accordingly, the supramolecular nanoparticles are rapidly dissociated. The Pt^IV^ prodrug is converted to chemotherapeutic cisplatin. Cisplatin activates two enzymes (nicotinamide adenine dinucleotide phosphate oxidase and superoxide dismutase), which generate H_2_O_2_ from O_2_ in tumor tissue. Thus, this system is effective through a self-augmented cascade.

### 5 CyD-based nanoarchitectures to promote photothermal therapy

In photothermal therapy, light energy is converted to thermal energy, which ultimately kills malignant cells [86]. In general, tumors are less resistant to heat than normal tissues. Advantageously, even light of long wavelengths is directly employable. Furthermore, this method is less affected by drug resistance and side-effects. A hydrogel was prepared by using both the electrostatic self-assembly between graphene oxide and a quaternized polymer and the formation of a pseudopolyrotaxane between α-CyD and poly(ethylene glycol) monomethyl ether (many α-CyD molecules were threaded into the polymer chains) [87]. NIR light (808 nm) was absorbed by graphene oxide and converted into heat for photothermal therapy. At the same time, the heat induces the gel–sol transition of the hydrogel to release the encapsulated drug which add to the photothermal effect for therapy.

**Figure 8:**
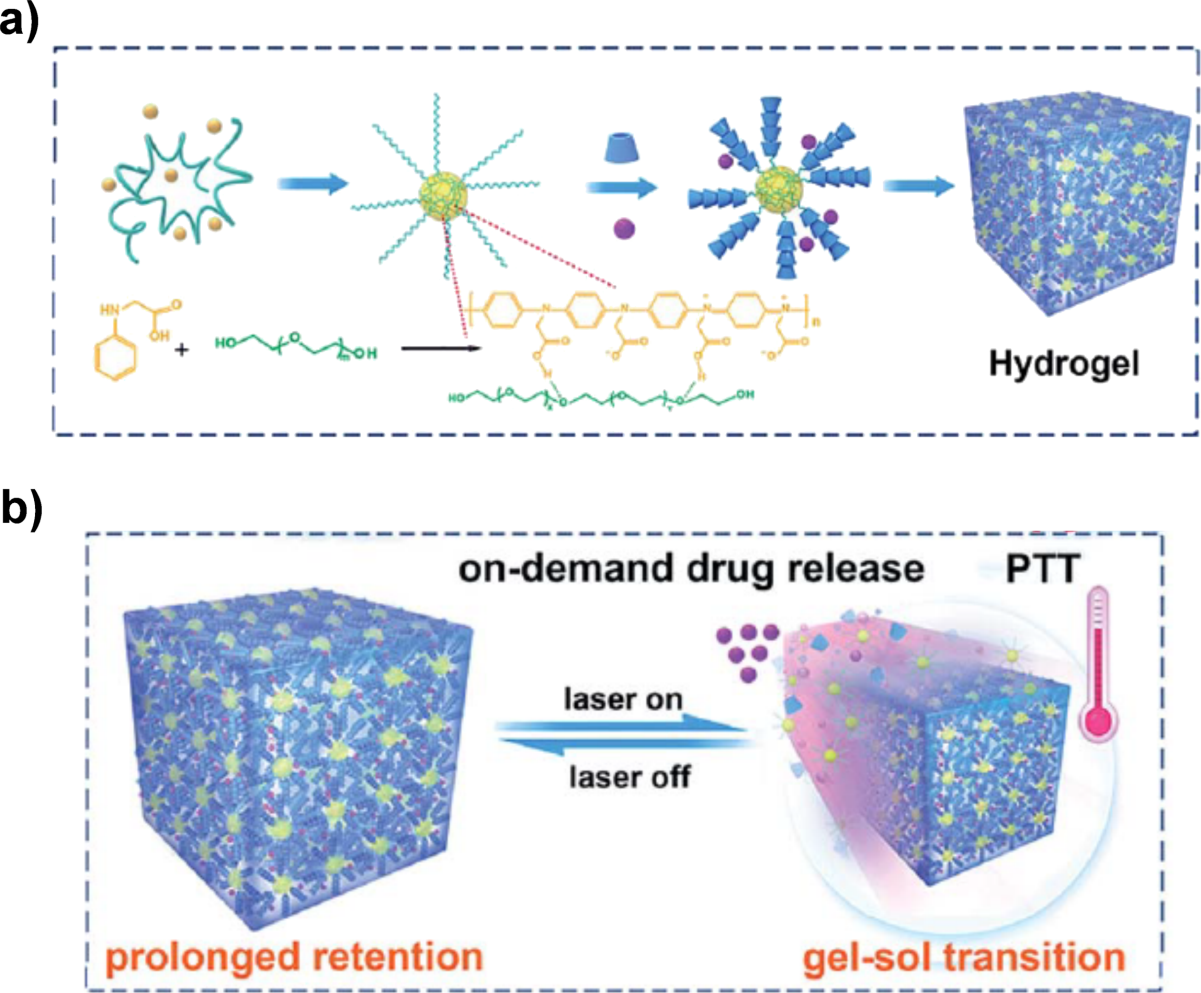
(a) Hydrogel production through pseudopolyrotaxane formation of poly(ethylene glycol) with α-CyDs. (b) Photothermal effect induced by the irradiation with NIR-II light (1064 nm laser). Simultaneously, a gel–sol transition occurred to release the drug (purple circles), promoting therapeutic efficiency. Figure 8 was adapted from [88] (“NIR-II light-modulated thermosensitive hydrogel for light-triggered cisplatin release and repeatable chemo-photothermal therapy“ by C. Ruan et al., *Chem. Sci.*, vol 10, issue 17. © The Royal Society of Chemistry 2019, distributed under the terms of the Creative Commons Attribution-NonCommercial 3.0 Unported License, https://creativecommons.org/licenses/by-nc/3.0). This content is not subject to CC BY 4.0.

Even NIR-II light (1000–1400 nm) is usable. In Figure 8, poly(ethylene glycol) chains (green) were tethered through hydrogen bondings to poly(*N*-phenylglycine) (yellow), which serves as the NIR-II absorber [88]. Upon the addition of α-CyDs, a hydrogel was formed through polyrotaxane formation of poly(ethylene glycol) with α-CyDs, in which adjacent α-CyDs interact to provide physical crosslinkings. Cisplatin (purple circle) was loaded in the hydrogel. Under NIR-II laser irradiation, the localized photothermal effect directly ablates thermosensitive cancer cells. At the same time, the temperature elevation by the photothermal effect induces gel–sol transition of the nanomedicine, which the release of encapsulated cisplatin from the hydrogel.

Mitochondria-targeting photothermal therapy was accomplished by two-dimensional nanoassemblies, prepared from β-CyD-grafted graphene oxide and transferrin (tumor-targeting protein) which bears poly(ʟ-lysine), mitochondrion-targeting peptide, poly(ethylene glycol), and arylazopyrazole (*trans* isomer) [89]. Under irradiation with NIR light (808 nm), the photothermal effect disrupted mitochondrial function, leading to inhibition of tumor growth.

### 6 Some recent topics on medical applications of CyD

#### 6.1 Chemical modification of CyD for precise targeting to predetermined cells

A multicharged nanoassembly was constructed from β-CyD bearing seven hexylimidazolium units, adamantane-grafted hyaluronic acid, and chlorambucil (an anticancer drug) [90]. In cancer cells, the β-CyD composite was disassembled by the enzyme hyaluronidase and released the hexylimidazolium-modified β-CyD to form a stable 1:1 complex with ATP in the cell. As the result, the ATP-mediated pump function of P-glycoprotein for the removal of foreign molecules was suppressed. The therapeutic efficacy of chlorambucil was greatly increased since it remained in the cell for a long time. Furthermore, an anticancer drug and a resistance-suppressing gene were simultaneously delivered for cooperation [91]. For delayed drug release, anionic β-CyD polymers bearing carboxylate residues are useful [92]. Metal complexes of CyD [93] and metal-organic frameworks (MOFs) containing CyDs [94–96] were also developed for DDSs.

By combining a thermoresponsive poly(*N*-isopropylacrylamide) star polymer with a β-CyD core, adamantane-terminated poly(ethylene glycol) polymer, and α-CyD, a supramolecular hydrogel was synthesized [97]. The hydrogel involved two types of supramolecular self-assemblies (inclusion complex formation between β-CyD and adamantane and polyrotaxane formation between α-CyD and poly(ethylene glycol) chains). When the composite enters human body, the temperature increase from 25 to 37 °C enhances the hydrophobic interactions of the poly(*N*-isopropylacrylamide) segments, stabilizing the hydrogel. The anticancer drug DOX encapsulated in the hydrophobic core is slowly released through the dissolution of the hydrogel to micelles.

By modifying β-CyD with both *N*-acetyl-ʟ-cysteine and arginine, insulin was orally delivered [98]. *N*-Acetyl-ʟ-cysteine enhances the mucoadhesion of the drug to enhance its biological absorption, whereas arginine promotes the intestinal absorption of insulin. The composite was sufficiently water-soluble and applicable to the oral delivery of insulin, which is mostly administered at present through subcutaneous injection. Moreover, a glutamine–β-CyD conjugate was used to deliver DOX for the treatment of triple-negative breast cancer, which is difficult to cure [99]. By combining β-CyD and poly(2-ethyl-2-oxazoline), DOX was released in response to both the reduction by glutathione and the pH decrease [100]. In order to deliver DOX to the cell nuclei, water-soluble conjugates of β-CyD with C_60_ fullerene have been employed [101].

CyDs are also useful for the treatment of COVID-19, which has been spreading all over the world as pandemic [102–104]. For example, remdesivir is a promising antiviral drug for the treatment of COVID-19. However, this compound is poorly soluble in aqueous media and chemically unstable. In order to solve these problems, CyDs were employed as additives [105–106]. This is an extension of classical applications of CyD. Alternatively, the complex of CyD with soluble ACE2 (i.e., the extracellular region of the ACE2 receptor on human cells) was developed [107]. The SARS-CoV-2 virus enters human cells through the binding of its spike to ACE2. Hence, the soluble ACE2 competitively suppresses the viral infection. The primary role of CyD here is also to increase the solubility of the protein through the binding to bulky and apolar side chains. The antiviral activity of modified CyDs towards various viruses has been previously reported as well [108].

#### 6.2 Molecular imprinting on CyD to bind large drugs

It often happens that one CyD molecule is too small and insufficient to bind large drugs. In these cases, molecular imprinting is a useful and convenient method [109–110]. In this method, CyD molecules (or their derivatives) are polymerized in the presence of a target compound (template). In the course of molecular imprinting, the solution structure involving the bindings of CyD molecules to the template is frozen in the polymer such that multiple CyD molecules are placed in the polymer complementarily to the large drug. Although each of these CyD molecules in the molecularly imprinted polymer binds only a small part of the large drug, these CyD molecules are arranged in a predetermined position (and orientation) by molecular imprinting. Thus, their assembly as a whole binds the large drug strongly and selectively. Many CyD-based carriers of drugs were prepared by means of this methodology and used for DDSs [111]. For example, ʟ-DOPA was delivered to a target site through encapsulation into a molecularly imprinted β-CyD polymer [112]. This prodrug for Parkinson’s disease is easily degraded when exposed to water or light but stably protected in the polymeric structure. Alternatively, angiotensin II as an octapeptide hormone for blood pressure regulation (Asp-Arg-Val-Tyr-Ile-His-Pro-Phe) was precisely recognized by a molecularly imprinted β-CyD polymer [113]. Surprisingly, a difference of only one amino acid is clearly differentiated. Apparently, the solution conformation of the oligopeptide, rather than its primary structure, is recognized by the molecularly imprinted β-CyD polymer. By extending this concept, a pH-responsive hydrogel was prepared [114].

## Conclusion

CyDs are easily obtainable at low cost and show many unique properties required for the application to medicinal purposes. CyDs are characterized by (1) a preorganized three-dimensional molecular structure of nanometer-size, (2) easy chemical modification to introduce functional groups, and (3) the formation of dynamic inclusion complexes with various guests in water. There are only few systems that exhibit all these properties simultaneously.

Through recent developments in nanoarchitectonics, these excellent properties of CyDs have been further extended to the construction of well-designed nanostructures for advanced drug delivery systems (DDSs). In some nanoarchitectures, their physicochemical and biological properties are successfully modulated by using CyDs as molecular frameworks with both structural restraint and reasonable flexibility. In other nanoarchitectures, reversible internal joints are formed through inclusion complex formation of CyDs with appropriate components (of course, the inclusion process can be directly used to load drugs). Advantageously, various functional groups are introducible to desired positions in the nanoarchitectures through chemical modifications of CyDs, facilitating a precise design for predetermined DDSs. Inclusion complexes of CyDs are sufficiently dynamic so that the drugs encapsulated in CyD-based nanomedicines are released at desired timing with the use of external stimuli. Furthermore, even multiple drugs can be simultaneously delivered to target sites with precise spatiotemporal control. Sequential delivery of multiple drugs should be also possible by appropriate molecular design. Undoubtedly, CyD-based nanoarchitectures should be essential and indispensable for further advancements of DDSs and relevant areas.
